# The Enigma behind Pituitary and Sella Turcica

**DOI:** 10.1155/2015/954347

**Published:** 2015-06-23

**Authors:** Umarevathi Gopalakrishnan, Lodd Mahendra, Sumanth Rangarajan, Ramasamy Madasamy, Mohammad Ibrahim

**Affiliations:** ^1^Department of Orthodontics and Dentofacial Orthopaedics, Sri Venkateswara Dental College and Hospital, Thalambur, Chennai 600130, India; ^2^Department of Prosthodontics and Crown and Bridge, Sri Venkateswara Dental College and Hospital, Thalambur, Chennai 600130, India

## Abstract

The pituitary gland's role as a functional matrix for sella turcica has not been suggested in orthodontic literature. This paper is an attempt to correlate the role of pituitary gland in the development of sella turcica. A case report of dwarfism associated with hypopituitarism is presented to highlight the above hypothesis.

## 1. Introduction

The functional matrix theory proposes that the origin, development, and maintenance of all skeletal units are secondary, compensatory, and mechanically obligatory responses to temporally and operationally prior demands of related functional matrices [1]. As per the theory, the skeletal unit's biomechanical role is to protect and/or support its specific functional matrix. When this functional matrix grows or is moved, the related skeletal unit responds appropriately to this morphogenetically primary demand. Sella turcica, meaning Turkish chair in Latin, is a saddle shaped depression in the sphenoid bone which holds the pituitary gland. There are literature reports of larger sella turcica in hyperfunctioning pituitary and smaller ones in hypofunctioning pituitary [2–15]. The probability of the pituitary serving as the functional matrix of sella turcica can be hypothesised based on this. We analysed this in detail by reporting the case of a hypofunctioning pituitary gland.

## 2. Case Report

**Figure 1:**
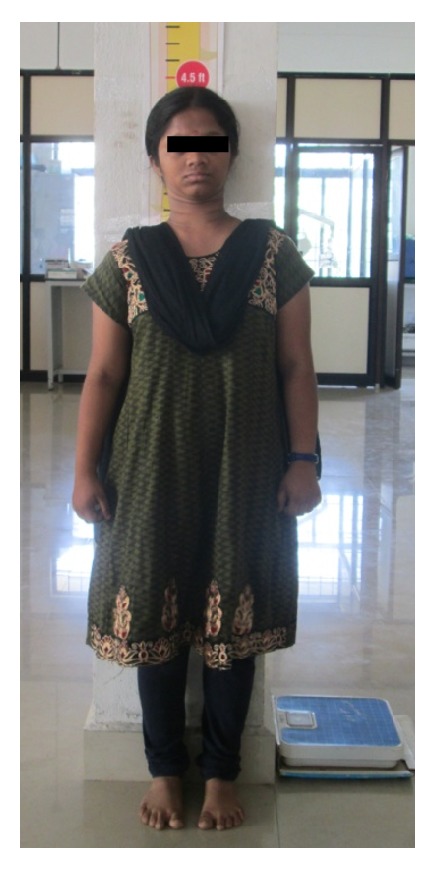
Height of the patient.

**Figure 2:**
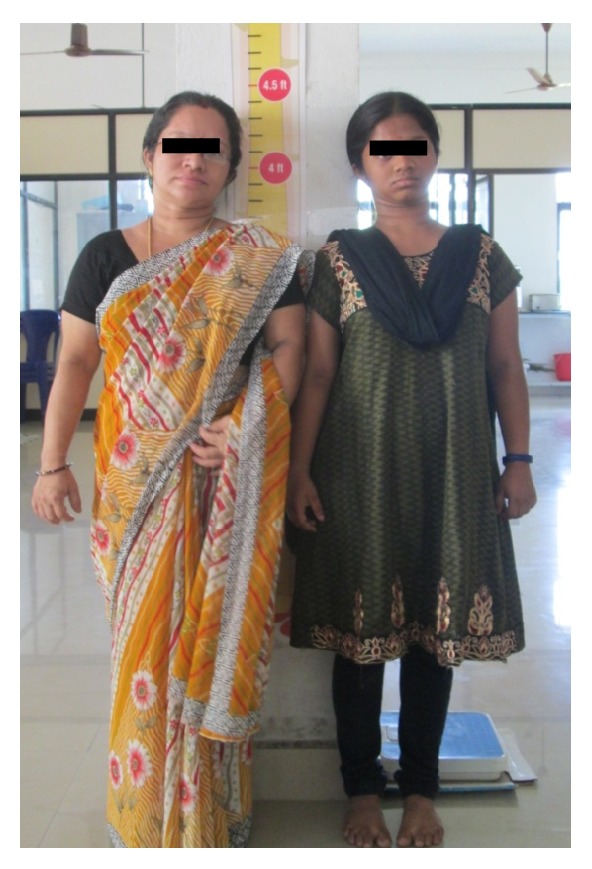
Patient and her mother of similar height.

**Figure 3:**
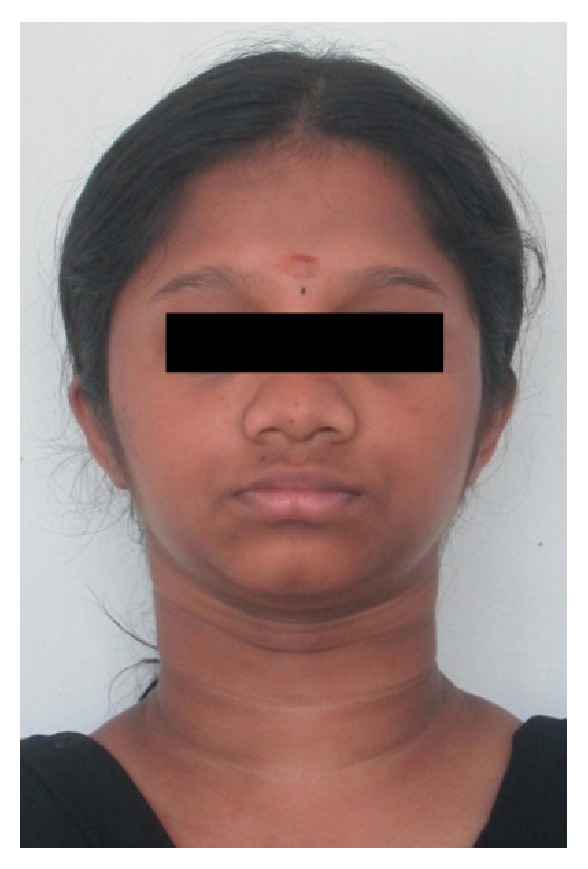
Extraoral photographs.

**Figure 4:**
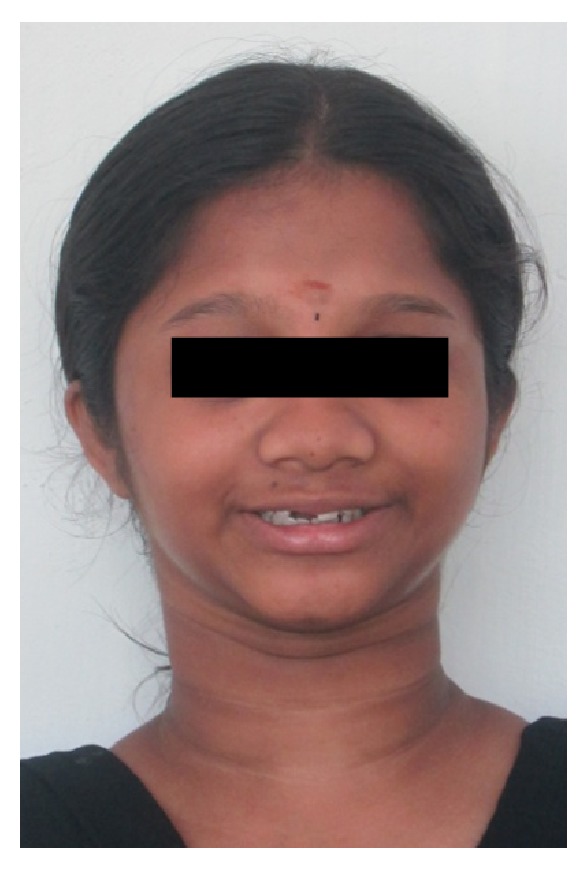
Extraoral photographs.

**Figure 5:**
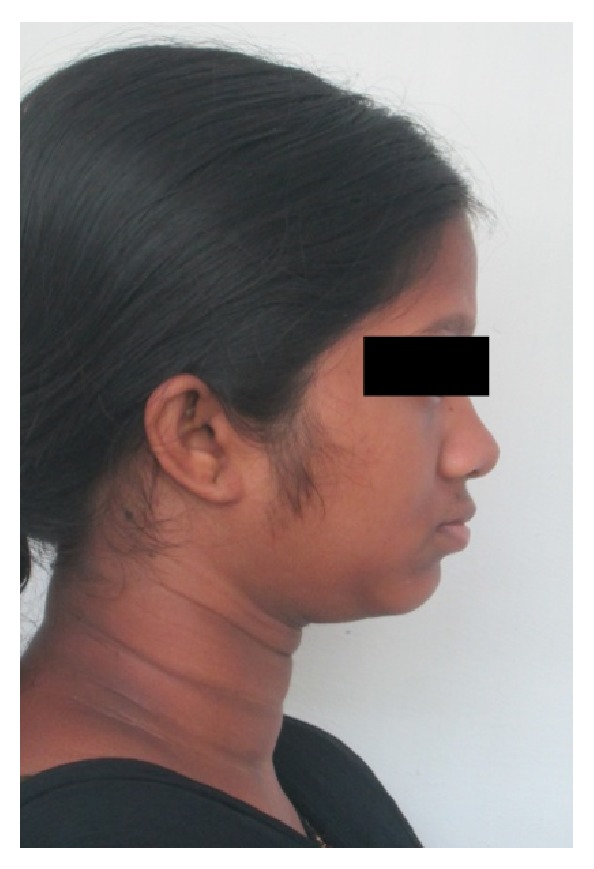
Extraoral photographs.

**Figure 6:**
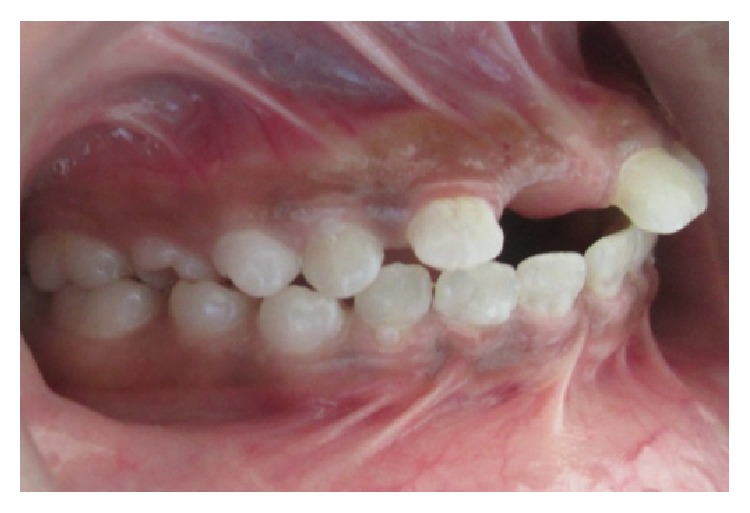
Intraoral photographs.

**Figure 7:**
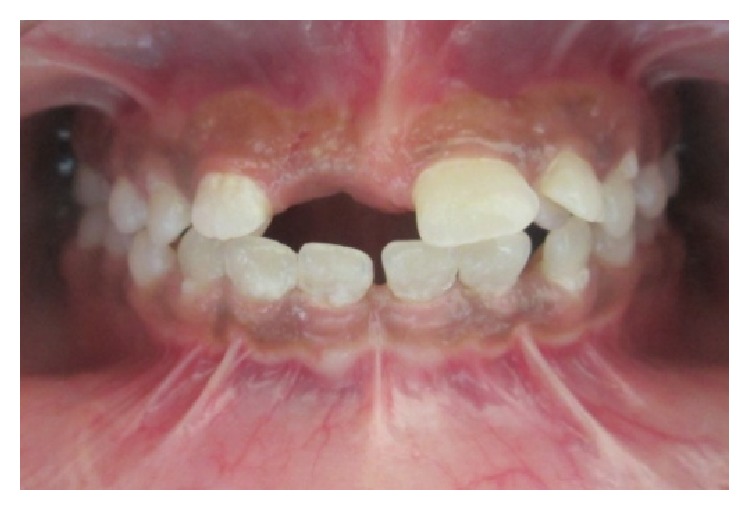
Intraoral photographs.

**Figure 8:**
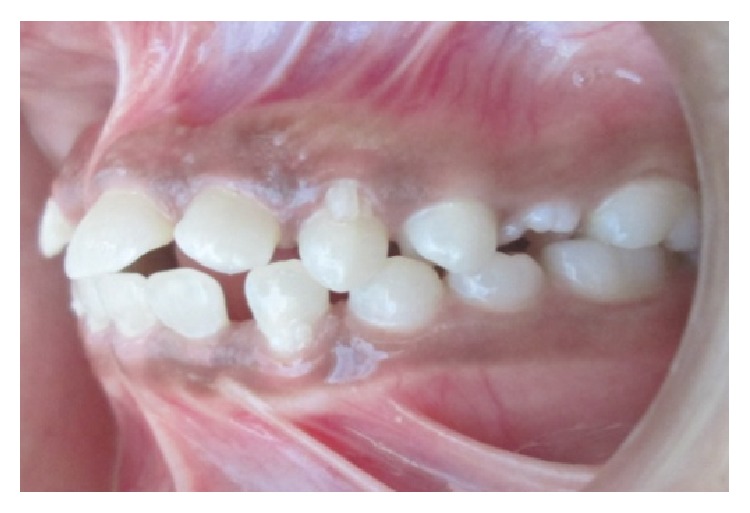
Intraoral photographs.

**Figure 9:**
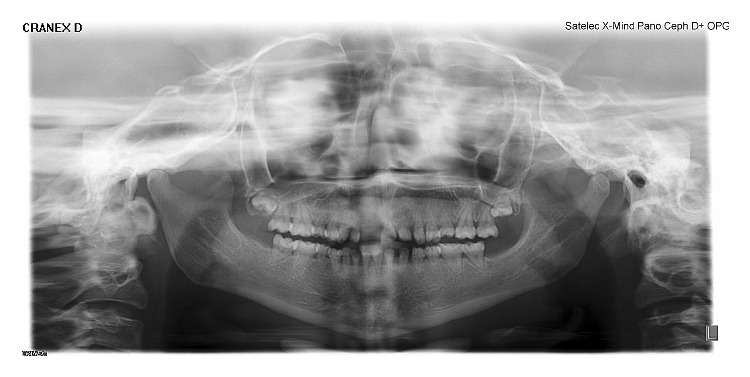
OPG of the patient revealing short clinical crown with conical roots.

**Figure 10:**
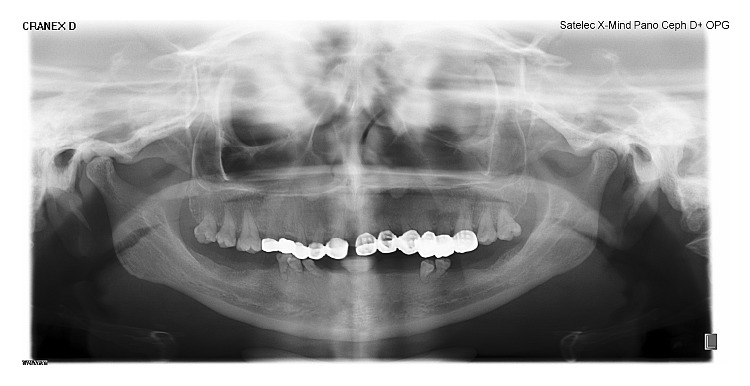
Mother's OPG revealing short conical roots.

**Figure 11:**
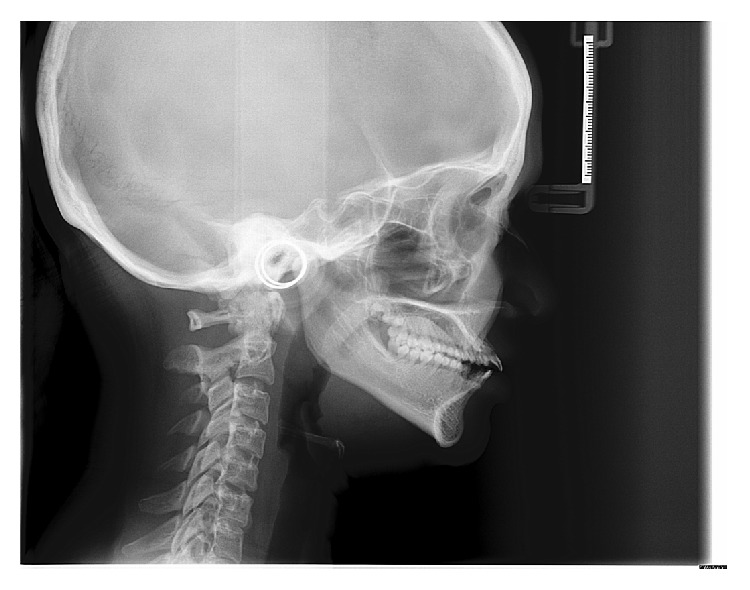
Lateral cephalogram of the patient.

A 15-year-old female reported to our college with complaint of missing front tooth. She was a dwarf with a height of 4.5 feet and a weight of 31 kg (Figure 1). History revealed that she was diagnosed as a case of hypopituitarism at 3 years of age. Her mother had a similar condition (Figure 2) and all her siblings were reported to be normal. Her basal growth hormone levels were 0.30 ng/mL at 3 years of age and 2.10 ng/mL at 11 years of age as per the records produced by her. Growth hormone replacement therapy was suggested for the patient by her endocrinologist which she had not taken. Her profile was mildly convex with competent lips (Figures 3, 4, and 5). She had class I molars with proclined incisors and generalised spacing. She gave a history of trauma a few months back leading to the loss of her upper left central incisor (Figures 6, 7, and 8). She had generalised microdontia with short conical roots as revealed by the OPG (Figure 9). Her mother's OPG (Figure 10) too revealed microdontia which is consistent with the dental findings of congenital hypopituitarism [16]. Her lateral cephalogram revealed a class I skeletal base (Figure 11 and Table 1). A striking feature in the lateral cephalogram was the decreased sella size (Table 2). The size of sella turcica assessed from radiographs typically ranges from 4 to 12 mm for the vertical and from 5 to 16 mm for the anteroposterior dimensions [17–20]. The lower limits of normal for depth and length of sella on radiographs are 4 mm and 5 mm [21]. In our case on superimposing the sella tracing over a graph sheet with millimeter readings [18], the vertical dimension was found to be 3.5 mm and anteroposterior dimension was 4 mm. The dimensions in our case are lower than the normal lower limit.

Since the patient's main complaint was missing incisor, she was not interested in getting orthodontic treatment despite having proclined incisors. Hence, no orthodontic treatment was carried out for this patient.

## 3. Discussion

For the pituitary to serve as the functional matrix for the sella, three things should be considered. (1) The pituitary should be formed before the cartilaginous sella. (2) Any growth increase of sella must follow that of pituitary. (3) Any abnormal growth morphology of pituitary should be reflected in the sella as well.

Sheng and Westphal [22] and Kjær and Fischer-Hansen [23] found that the pituitary gland develops before the cartilaginous sella turcica has been formed. O'Rahilly and Müller [24] reported that the hypophyseal cartilages fuse around the existing hypophysis to form the body of the sphenoid bone containing the hypophyseal fossa. The thyroid stimulating hormone is secreted by the pituitary around the 15th week of intrauterine life. It is at this time that the cartilaginous precursor of the sella is first noticed in the foetus [25]. This fact that the pituitary gland starts functioning even before the cartilaginous precursor of the sella is being formed favours our hypothesis.

Changes in the sella turcica during growth in childhood have been studied radiographically by Björk and Skieller [26] and histologically by Melsen [27] which showed that the sella turcica increases in size during childhood. The increase occurs as a result of resorption at the interior wall of the dorsum sella. Age related increase for both genders was reported by Axelsson et al. [9]. For the pituitary to serve as the functional matrix of sella, age related increase in pituitary gland should be the preceding factor before an increase of size in sella. Argyropoulou et al. [28] through their retrospective MRI study stated that an age related increase of sella turcica size is expected because its contents, that is, the hypophysis, have been shown to increase in size with age. Siverman [18] reported that sella area increased with age possibly related to the function of the anterior lobe of pituitary.

The functional matrix's decrease/increase in function will have the reflection in the corresponding skeletal unit which is evident from the response of the alveolar bone and teeth. The tooth being the functional matrix of the alveolar bone, the alveolus forms as the tooth is erupting and regresses after tooth loss. A similar correlation can be seen with pituitary and sella turcica. The literature reports reveal that whenever pituitary gland enlarges there is a corresponding increase in size of sella and vice versa [2–15]. The radiological diagnosis of an enlarged sella turcica has been found to be associated with pituitary tumours (adenomas, meningioma, prolactinoma, and craniopharyngioma), cystic lesion (Rathke's cleft cyst and mucocele), aneurysm, pituitary hyperplasia (primary hypothyroidism), acromegaly, gigantism, and Nelson syndrome [2–8]. An enlargement of the pituitary gland with a corresponding increase in sella is noted in these cases.

A decrease in sella size was noted in hypofunctioning pituitaries. An abnormally small sella turcica was found in primary hypopituitarism [10–12], growth hormone deficiency [13], Williams's syndrome [29], and Cushing's syndrome due to adrenocortical adenoma [14]. The sella size is more affected if the onset of pituitary hypofunction is before the age of 6 years [15]. Our case is a similar condition where the hypopituitarism has caused a decreased size of the sella turcica. In a syndrome called Sheehan's, the pituitary gland undergoes necrosis due to infarction after a complicated delivery. In all such cases, a smaller sella turcica is noted [30–32]. Bakiri et al. [33] reported that the size of the pituitary residue in Sheehan's syndrome never exceeded one third of the normal pituitary gland and the size of the sella was significantly smaller when compared to controls.

## 4. Conclusion

All these literature reports and reviews strongly suggest a correlation between sella turcica and pituitary gland. They lend credence to the fact that pituitary gland could be serving as the functional matrix for the skeletal unit of sella turcica since it forms before the cartilaginous skeleton of sella and any morphological change in pituitary has a corresponding one in sella. But further tissue level investigations may be needed to validate these claims.

## Figures and Tables

**Table 1:** 

Cephalometric norms	Patient's values
SNA	74°
SNB	74°
ANB	0°
N Pr to A	−12 mm
N Pr to Pg	−19 mm
SN-Md plane	39°
FMA	30°
U1 to SN	126°
L1 to Md plane	100°

**Table 2:** 

Sella dimension	Norms	Patient's values
Vertical	4 to 12 mm	3.5 mm
Anteroposterior	5 to 16 mm	4 mm
